# Metabarcoding Reveals Impact of Different Land Uses on Fungal Diversity in the South-Eastern Region of Antioquia, Colombia

**DOI:** 10.3390/plants12051126

**Published:** 2023-03-02

**Authors:** Raul Aranguren, Samuele Voyron, Fabrizio Ungaro, Julio Cañón, Erica Lumini

**Affiliations:** 1 GAIA Research Group, Universidad de Antioquia, Medellín 050010, Colombia; 2 Department of Life Sciences and Systems Biology, University of Turin, 10124 Turin, Italy; 3 Institute for Sustainable Plant Protection (IPSP), National Research Council (CNR), 10125 Turin, Italy; 4 Institute for Bio-Economy (IBE), National Research Council (CNR), 50018 Florence, Italy

**Keywords:** Colombian Andosols, fungal ITS2 metabarcoding, response ratio, alpha diversity, relevant fungal taxa

## Abstract

Changes in soil fungal communities caused by land use have not been sufficiently studied in South American Andosols, which are considered key food production areas. Since fungal communities play an important role in soil functionality, this study analysed 26 soil samples of Andosols collected from locations devoted to conservation, agriculture and mining activities in Antioquia, Colombia, to establish differences between fungal communities as indicators of soil biodiversity loss using Illumina MiSeq metabarcoding on nuclear ribosomal ITS2 region. A non-metric multidimensional scaling allowed to explore driver factors of changes in fungal communities, while the significance of these variations was assessed by PERMANOVA. Furthermore, the effect size of land use over relevant taxa was quantified. Our results suggest a good coverage of fungal diversity with a detection of 353,312 high-quality ITS2 sequences. We found strong correlations of Shannon and Fisher indexes with dissimilarities on fungal communities (r = 0.94). These correlations allow grouping soil samples according to land use. Variations in temperature, air humidity and organic matter content lead to changes in abundances of relevant orders (Wallemiales and Trichosporonales). The study highlights specific sensitivities of fungal biodiversity features in tropical Andosols, which may serve as a basis for robust assessments of soil quality in the region.

## 1. Introduction

Changes in the structure of soil fungal communities are important indicators of variation in soil health caused by land use. Furthermore, soil functions, related to nutrient cycling, ecosystem provisioning and climate regulation can decrease due to loss of fungal diversity [1,2]. Although there is information on the effect of different land uses on tropical soil fungal communities [3,4,5], the distribution of fungal taxa, functional groups and biogeographical patterns, at local scale in Colombian soils, is still poorly and discontinuously analysed [6]. Specifically, Andosols from the central Andes mountain range in Colombia are relevant units of agricultural productivity, complex microbial habitats and important water reservoirs due to their high spatial variation in phreatic zones, thickness of organic horizons and effective cation exchange capacity [7]. However, information is lacking in understanding how human activities affect the diversity and structure of fungal communities in Colombian Andosols. The assessment of local soil fungal communities, whose composition depends on the characteristics of the bioclimatic region and the specific conditions of each site, is the basis of studies on their ecological behaviour [8]. Therefore, the identification of fungal taxa associated with soils is necessary for understanding these complex communities [9]. In addition, assessing the effect of specific land uses on individual soil microbial taxa is key to propose management practices of soil microbial diversity to maintain or increase soil quality [10,11].

Metabarcoding based on sequences of the internal transcribed spacer region (ITS) for the identification of fungal taxa have been recognised as a universal barcode able to detect poorly sequenced taxa in samples of different nature [12,13]. Characterisation of fungal communities using metabarcoding of ITS region sequences has expanded the capability to identify the effects of land uses in shaping these communities across space and time, associated to factors such as leaf litter, soil nutrients, pH and organic matter content [14,15,16]. Tropical soils are important for the study of fungal ecological preferences and their driving factors, considering that the diversity of fungal groups can peak in tropical ecosystems [17].

Previous studies have obtained ITS amplicon pools in soils retrieved from tropical ecosystems, such as rainforest, dry forest, littorals, coasts and the Andean forests [18,19,20]. Moreover, shifts of fungal dominant taxa have been reported, along with the increase of facultative fungal abundances in deforested areas [21] and the decline of fungal richness and diversity indexes, due to increments of phosphoric fertilisation rates [22]. Positive correlations between pathogenic fungal distribution and soil fertility factors have been reported as well [23].

In this study, we focused on the characterisation of fungal communities of tropical Andosols, by an Illumina metabarcoding approach that targets the ITS2 region, a globally accepted barcode for fungi [24]. The impact on fungal communities was assessed on three different types of land use with different levels of degradation, in the south-eastern region of Antioquia (Colombia): (1) natural forest areas; (2) areas under weak pressure by agriculture activities; and (3) areas subject to considerable pressure by mining activities related to clay extraction. We compared features of fungal communities to establish the effect size of land use on soil biodiversity. We characterised fungal communities from Colombian Andosols, testing how features and keystone taxa of soil fungal communities vary with each land use perturbation in this selected area. Our hypothesis is that changes in features of fungal communities can be useful to identify and group soils according to the intensity of degradation processes. In the frame of an increasing demand to understand the factors driving soil fertility and resilience, the results provided useful information on key fungal components to be further exploited as indicators in a sustainable ecosystem management perspective.

## 2. Results

### 2.1. Sequencing and Soil Fungal Community Composition

In total, 353,312 high-quality ITS2 sequences, averaging 11,330 sequences per sample, were retained. One sample from the quarry clays of La Ceja (LC-QC2) was discarded due to the low number of retrieved sequences (less than 1000 reads). Despite the high number of valid sequences, the land use associated with mining activities areas (MEA) presented a lower number of different operational taxonomic units (OTUs). On the contrary, natural forest areas (NFA) and agricultural activities areas (AGA) showed the highest number of OTUs (Table S1). A total of 494 OTUs, 9 phyla, 29 classes, 53 orders, 117 families, 158 genera and 174 species were identified. Rarefaction curves built on the rarefied OTU table suggest that a plateau was reached (Figure S1), indicating that the Illumina metabarcoding approach revealed a good representation of the global fungal composition in every specific activity. Phylum Basidiomycota was dominant in NFA (47.20%) and MEA (69.37%) land uses, whereas Ascomycota was dominant in AGA (36.33%) and NFA (44.40%).

**Figure 1:**
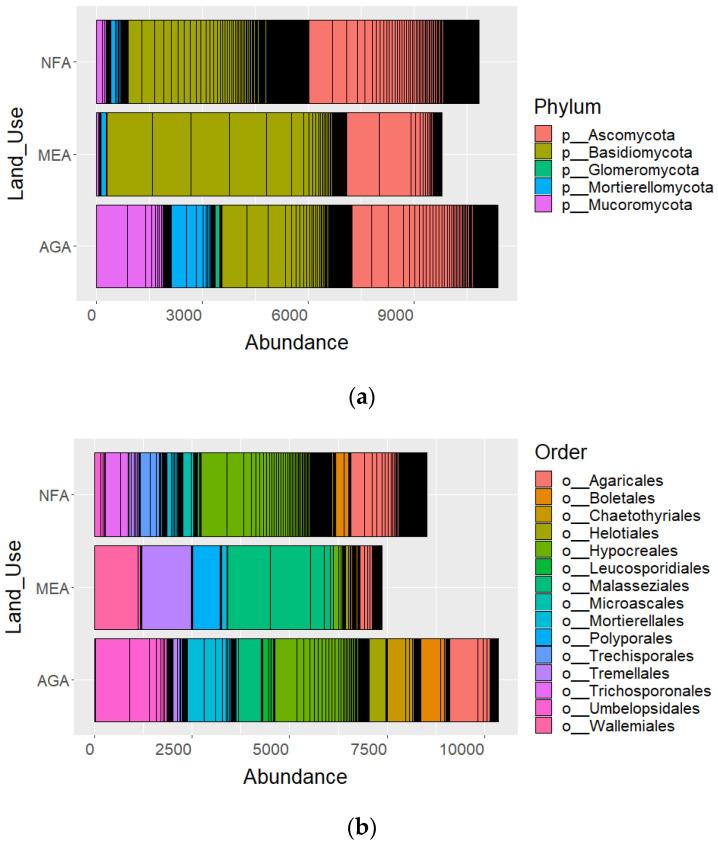
Abundances of fungal dominant taxa in natural forest areas (NFA), agricultural activities areas (AGA) and mining extraction activities (MEA): (**a**) abundances at phylum level; (**b**) abundances at order level.

Hypocreales was the most abundant order in NFA (3303 reads) and AGA (2412 reads). Malasseziales was the order with higher abundance observed in MEA (2707 reads). Nevertheless, this order was scarcely observed in NFA (12 reads) (Figure 1). The chi-square test showed that the OTU abundance of Basidiomycota was significantly higher in MEA, followed by NFA. Furthermore, we found that OTU abundance of Ascomycota was significantly higher in NFA and AGA land uses (Table 1). At order level, the chi-square test showed that OTU abundance of Hypocreales was significantly higher in NFA than in AGA (*x*^2^ = 268.57; *p* ≤ 0.01) and MEA (*x*^2^ = 2142.47; *p* ≤ 0.01), as well as in AGA than in MEA (*x*^2^ = 1166.44; *p* ≤ 0.01). Likewise, test results highlighted that the abundance of OTU belonging to Malasseziales order was significantly higher in MEA land use than in NFA (*x*^2^ = 4173.38; *p* ≤ 0.01) and AGA (*x*^2^ = 579.36; *p* ≤ 0.01).

Among the 494 OTUs, 223 belonged to Basidiomycota, 164 to Ascomycota, 25 to Mucoromycota, 17 to Mortierellomycota, 16 to Glomeromycota and 41 to unassigned phyla. As for the different land uses, 121 taxa were observed in NFA, 72 in AGA and 48 in MEA. The land use with the highest number of prevalent species was NFA (12 species) followed by AGA (4 species) and by MEA (1 species). *Metarhizium anisopliae* (Metchnikoff) and *Saitozyma* sp. were the most frequent species in NFA, whereas *Trichoderma asperellum* (Samuels, Liechfeldt et Nirenberg) was the most frequent in AGA. *Agrocybe pediades* ((Fr.) Fayod) was a prevalent species under AGA and MEA land uses in both weakly and highly degraded soils (Table 2).

### 2.2. Soil Fungal Community Variations among Land Uses

**Figure 2:**
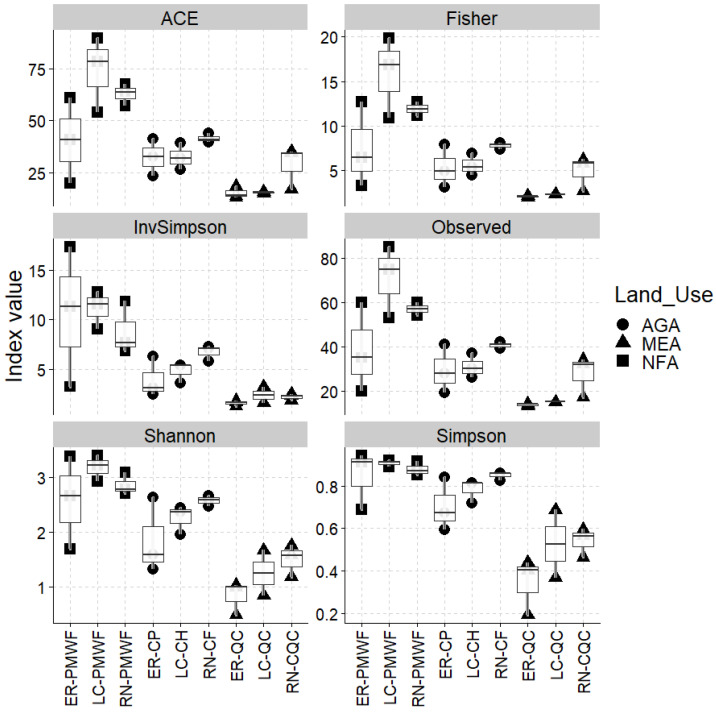
Alpha diversity indexes of Colombian Andosols fungal communities retrieved in El Retiro- Pre-montane wet forest (ER-PMWF); La Ceja-Pre-montane wet forest (LC-PMWF); Rionegro-Pre-montane wet forest (RN-PMWF); El Retiro-Forestry crops of *Pinus* sp. (ER-CP); La Ceja-Crops of *Hydrangea* sp. (LC-CH); Rionegro-Crops of *Fragaria ananassa* (RN-CF); El Retiro-Quarry clays (ER-QC); La Ceja-Quarry clays (LC-QC); Rionegro-Closed quarry clays (RN-CQC) (AGA = agricultural activities areas; MEA = mining extraction activities; NFA = natural forest areas).

The pairwise comparison of contrasting land uses ‘NFA vs. AGA’ and ‘NFA vs. MEA’ showed significant differences in terms of the composition of soil fungal communities. However, the fungal composition of the land uses ‘AGA and MEA’ was not significantly different at every taxonomic level analysed. R^2^ values were higher and PERMUTEST were not significant, considering the comparison at order level followed by species (Table S2). Nevertheless, land uses show variations in the alpha diversity indexes, with significant PERMANOVA differences observed among ‘NFA vs. AgA’ (R^2^ = 0.404, *p* = 0.002), ‘NFA vs. MeA’ (R^2^ = 0.746, *p* = 0.001) and ‘AGA vs. MeA’ (R^2^ = 0.580, *p* = 0.001). Higher alpha diversity values occurred in the NFA land use, while lower values occurred in MEA. The alpha diversity indexes showed consistent patterns among land uses (NFA > AGA > MeA) (Figure 2).

### 2.3. Fungal Community Responses to Variations in Soil Properties and Environmental Factors

The studied Andosols have an acidic to weakly acidic pH (range 5.3 to 5.7), with some properties, such as organic matter content and soil moisture, decreasing with increasing land degradation. On the contrary, soil electrical conductivity and total dissolved solids increase with the degree of degradation. Similarly, environmental parameters such as relative air humidity and barometric pressure decrease in degraded areas, while other parameters such as soil temperature increase in those areas. In AGA sites, pH and soil temperature decreased. However, significant differences were observed among land uses in soil properties such as soil electrical conductivity, soil moisture and organic matter content, as well as in environmental parameters, i.e., relative air humidity and soil temperature (Table S3).

**Figure 3:**
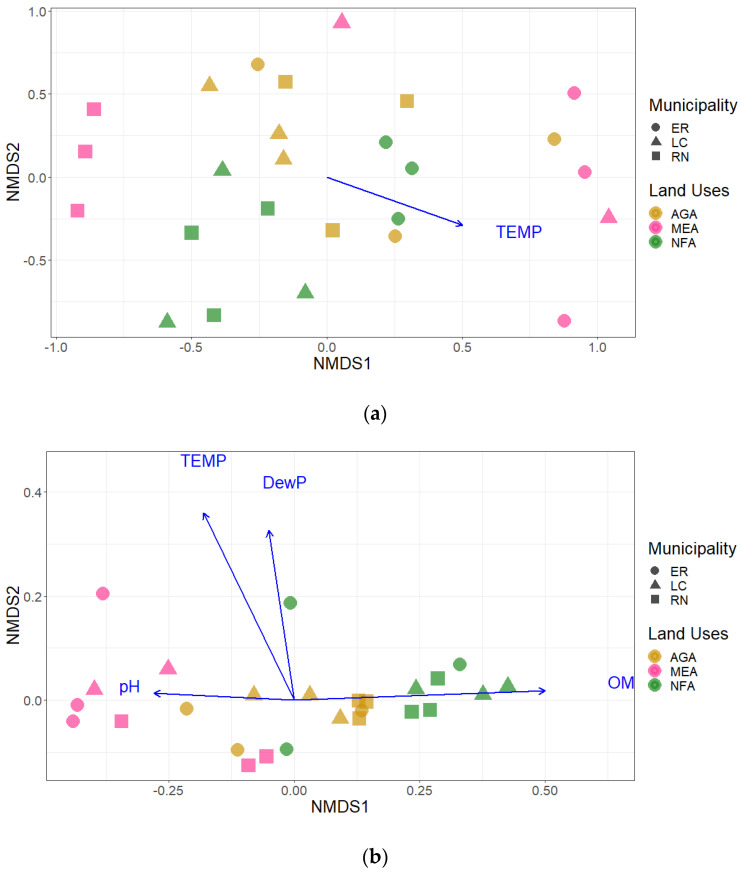
Non-metric multidimensional scaling of 26 soil samples with driver factors: (**a**) ordination based on order compositions (stress value = 0.243); (**b**) ordination based on alpha diversity index values (stress value = 0.027) (AGA = agricultural activities areas, MEA = mining extraction areas, NFA = natural forest areas, DewP = dew point temperature, OM = organic matter, TEMP = soil temperature).

The NMDS dissimilarity distance matrix with Jaccard’s coefficient based on fungal composition at order level did not show a clear gradient of samples according to land use, and only soil temperature was significatively correlated with sample ordination (Figure 3a). Nevertheless, fungal communities from NFA and AGA land uses were closely grouped and a gradient of sample position with land use was detected in the sample ordination based on alpha diversity indexes. A significant positive correlation between sample gradient and organic matter content was observed jointly with negative correlations with soil temperature, dew point temperature and pH (Figure 3b).

### 2.4. Effect Size

**Figure 4:**
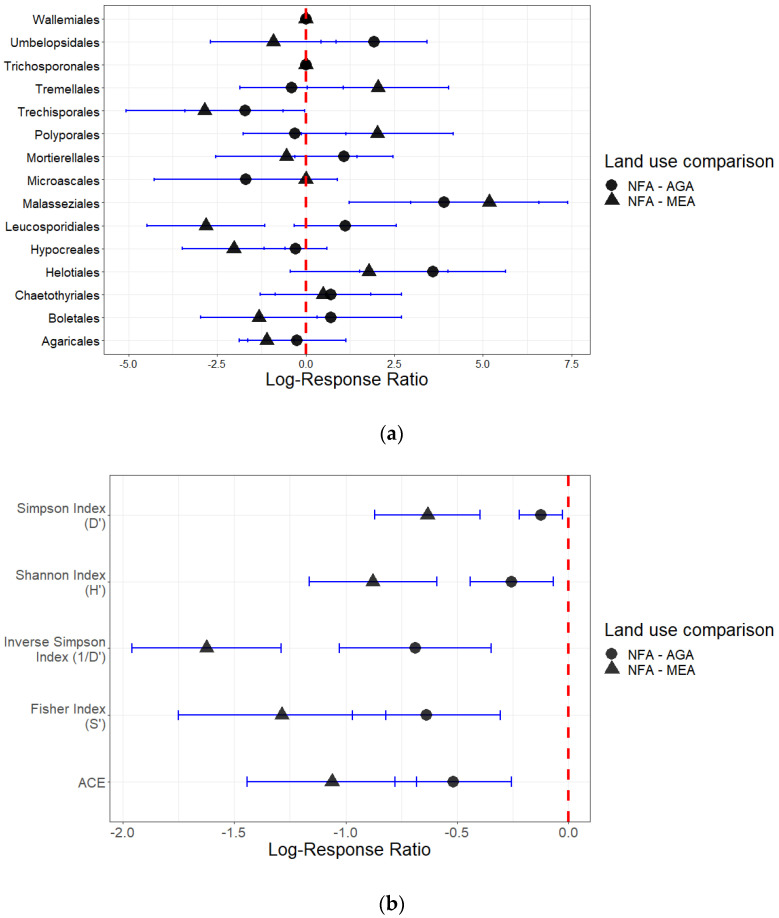
Log-response ratios (LogRR): (**a**) abundances of dominant order; (**b**) alpha diversity index values (AGA = agricultural activities areas, MEA = mining extraction areas, NFA = natural forest areas). If the 95% CI of LogRR did not overlap with 0, then responses were significant at *p* < 0.05 level.

Abundances of order Leucosporidiales, Trechisporales and Malasseziales were significantly affected by AGA and MEA land uses. Both land uses exerted a significant negative effect on alpha diversity indexes (Figure 4). The Fisher index (S’) has the largest effect size among contrasting land uses (LogRR NFA-AGA = −0.637; LogRR NFA-MEA = −1.285). Likewise, soil fungal community richness measured by means of alpha diversity indexes decreased with mean response ratio (MRR) of 0.443 and 1.096 in AGA and MEA land uses, respectively. MRR highlighted the effect size due to the abundances of dominant orders, with values of 1.177 in AGA and 1.539 in MEA. Wilcoxon rank sum test showed that the MRR between NFA and AGA was significantly different from the MRR between NFA and MEA (*p*-value < 0.05), indicating a lower impact in agricultural lands compared to mining.

## 3. Discussion

The impacts of land use changes on the composition of soil fungal communities have not been sufficiently studied in South American Andosols. As a consequence, datasets of fungal sequences, along with their metadata of samples from these areas are quite scarce [25]. In this frame, we compared different characteristics of fungal communities to assess the effect size of different land uses on soil biodiversity. The fungal communities were studied by means of a metabarcoding approach, testing how features and keystone taxa of soil fungal communities vary with each land use perturbation in this selected area.

This study was conducted to test the hypothesis that certain changes in fungal communities are significantly related to the level of soil degradation beyond expected changes due to location or climate. Therefore, the characteristics of these fungal communities may be useful for identifying and clustering soil samples according to their level of degradation.

The average of high-quality ITS2 sequences retention obtained was comparable with previous sequencing efforts of fungal communities in similar tropical soils. For example, ITS2-ITS4 barcode approach to fungal characterisation of soils from subtropical wet forest retained an average of 11,293 high-quality ITS sequences [19]. The high number of taxa found in our Andosols (174 species) contrasts with the number detected in similar soils and land uses located at lower altitudes. For instance, in Andean agricultural soils of Boyacá, Colombia (located at 2800 m a.s.l), only 143 taxa were identified among 6 phyla by a deep sequencing of the ITS1 barcode [6], whereas only 30 fungal taxa were identified by a ITS1-ITS2 barcode approach in Andosols from Mexican pine forest, corn fields and rosebush (located at 1300 to 1700 m a.s.l) [26]. Studies on fungal communities along altitudinal gradients suggest that altitude and related environmental factors, such as air humidity and soil temperature, affect fungal community structure [27]. In this regard, the observed influence of soil properties on the composition of fungal communities agrees with previous studies that reported a correlation between soil community composition and soil characteristics, such as nitrogen content, water retention, pH and cation exchange capacity [28].

In our study, Glomeromycota was a phylum rarely observed. However, among the few high-quality ITS2 sequences of this phylum, 10 taxa were identified. Previous fungal community characterisations in comparable environments have reported lower AMF diversity values. For example, Landinez-Torres et al. [6] tested in Colombian Inceptisols (located at 2800 m a.s.l) an ITS1 region amplification by primers BITS and B58S3, identifying only two orders for Glomeromycota phylum (among 8 taxa), two families (Glomeraceae and Acaulosporaceae) and two species (*Glomus mosseae* (Nicol. and Gerd.) and *Entrophospora infrequens* (I.R.Hall)) [29]. Sequences of phylum Basidiomycota were frequently detected, especially in mining areas. This result corroborates the findings of other authors, in which most fungi in tropical Andosols collected from mosaics of warmer forest-crops and degraded ecosystems (as fragmented forest and areas where vegetation was totally removed) belonged to phylum Basidiomycota [30,31]. In the mining areas sampled, for instance, *Malassezia* sp. was the most abundant species. The high abundance of the genus *Malassezia* in degraded areas could be related to low organic matter contents and consequently to soil acidification [32].

On the other hand, samples from NFA showed a high abundance of species belonging to the order Hypocreales (phylum Ascomycota). In NFA land use, factors such as higher air humidity, lower soil temperature, wild vegetation and the absence of pesticide applications can promote the abundance of this order. Previous studies have reported higher abundances of *Metarhizium* genus in conservation areas being positively mediated by temperate environments, wild vegetative cover, high humidity and hosting insects [33]. Likewise, *Fusarium solani* (Mart.) Sacc. abundances are robustly associated with factors such as high soil moisture and a presence of entomopathogenic nematodes [34]. Nevertheless, conditions observed in NFA, such as thick layers of litter, were favourable compared to the high prevalence of other taxa such as *Saitozyma* sp. (order Tremellales), since *Saitozyma* genus is a taxa strongly correlated with the edaphic incorporation of carbon in pre-montane wet forest and forestry crops of *Pinus* sp. [35]. In AGA land use, the high abundance of Ascomycota sequences was attributed to the prevalence of *Trichoderma asperellum* Samuels, Lieckf. and Nirenberg, a fungal species commonly present in agricultural areas or related with soil added with mineral fertilisers [36,37,38].

The results reported in this study also indicate significant and analogous effects of land use on soil fungal community composition. The NMDS sample ordinations showed a gradient with samples from undegraded areas clustered near samples from agricultural areas and substantially far away from samples of highly degraded mining areas. Furthermore, soils from native, undegraded areas, separated by distances between 20 km and 25 km (locations RN-PMWF to ER-PMWF; LC-PMWF), shared more similarities in fungal order composition than those from agricultural and mining extraction areas located in the same municipality, indicating that the changes respond to factors that go beyond expected spatial variations. The PERMANOVA R^2^ values suggest that the land use factor can explain between 10% and 12% of the variation observed in fungal order composition (contrasting NFA-AGA and NFA-MEA, respectively). Likewise, land uses explain important variations on alpha diversity index in the same contrasts (41% variation in NFA-AGA and 75% variation in NFA-MeA). The observations illustrate the correspondence between dissimilarities of fungal composition in samples with the effect size (LogRR) on dominant order abundances.

The land use gradient displayed in NMDS plots based on order abundances and alpha diversity index implied positive correlations between soil fungal diversity with organic matter content, and negative correlations with soil temperature. Most of these effects are induced by clearances of vegetation and partial removals of topsoil horizons from the sampled agricultural areas and mining sites, since clearances of vegetation results in a reduction of soil moisture and nutrients availability, as well as a significant increment in temperature [39,40]. As a matter of fact, the corresponding variations in soil temperature and organic matter contents observed in anthropogenically impacted areas determine an enlargement of the effect size observed in the abundances of order Malasseziales (LogRR = 5.178) and Trichosporonales (LogRR = −2.848). Wallemiomycetes (order Malasseziales), for instance, have been reported as a xero-tolerant fungi, highly abundant in dry environments [41]. Conversely, populations of representative taxa in temperate wet forest dominated by deciduous trees, such as *Apiotrichum scarabaeorum* (Middelhoven, Scorzetti and Fell) Yurkov and Boekhout (order Trichosporonales), decrease in soils with low organic matter contents [35].

In particular, the ordination model based on alpha diversity index displayed a sample gradient as a function of the intensity of soil degradation. The patterns obtained for every alpha diversity index among land uses indicated that fungal richness was higher in undegraded areas and decreased with the degree of anthropogenic impact. The land use effect size over Fisher index (S’) suggests that the primary features of the fungal community that are affected by land use are the ratio of the number of taxa and the abundance of those taxa. In the validation of those observations, the sequence abundances of the most relevant taxa (such as prevalent species and dominant order) were negatively affected by land use change, despite high average ITS2 high quality sequences in agricultural (12,486 sequences) and mining areas (15,029 sequences). Furthermore, species richness downturned from 116 species identified in natural forest areas, to 68 in agricultural areas and 47 species in mining areas. This consistent drop in alpha diversity indices are unlikely to be solely the result of spatial variations, but rather the consequence of changes in the fungal community composition mainly caused by the limited availability of nutrients from organic matter and increments of soil temperature due to the level of soil degradation.

## 4. Materials and Methods

### 4.1. Study Site and Characterisation of Land Uses

**Figure 5:**
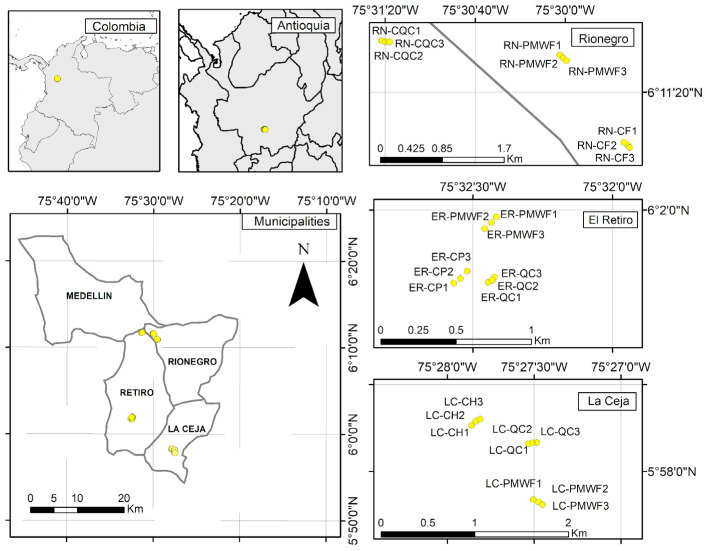
Geolocation of 27 soil samples retrieved in El Retiro-Pre-montane wet forest (ER-PMWF); La Ceja-Pre-montane wet forest (LC-PMWF); Rionegro-Pre-montane wet forest (RN-PMWF); El Retiro-Forestry crops of *Pinus* sp. (ER-CP); La Ceja-Crops of *Hydrangea* sp. (LC-CH); Rionegro-Crops of *Fragaria ananassa* (RN-CF); El Retiro-Quarry clays (ER-QC); La Ceja-Quarry clays (LC-QC); Rionegro-Closed quarry clays (RN-CQC) [45].

The field research took place in 2018 during a rainy season (September and October) in three municipalities (La Ceja, El Retiro and Rionegro), in the south-eastern region of Antioquia, Colombia (Figure 5). The municipalities are located in the central Andes Mountain range at altitudes between 2300 and 2700 m above sea level (m a.s.l). Pre-montane humid forest and mosaics of pasture-crops are typical ecosystems in the region, which exhibits a unimodal temperature cycle with an average temperature of 21 °C and an annual bimodal precipitation cycle with an average monthly precipitation of 387 mm. Within each municipality, samples of Andosols were collected in three land uses characterised by different intensities of land degradation, which were defined according to the fraction of topsoil removal [42]. Hence, soils from natural forest areas (NFA) were considered as reference of undegraded conditions, while soils retrieved in agricultural areas (AGA) with a topsoil removal between 10% and 20% were defined as weakly degraded, and soils of mining extractions areas (MEA) with a topsoil removal above 60% were considered as highly degraded.

A point-transect sampling was carried out along 100-m transects delineated from the centroid of the polygon encompassing each land use. Three equidistant points (at 50 m) were selected along each transects. Five subsamples (soil cores at 20 cm depth) were taken within a 2 m radius of each point to make a composite soil sample, using a soil auger with a diameter of 5 cm. Twenty-seven composite soil samples were collected and stored in plastic bags at 4 °C for further analyses. Environmental variables (soil temperature, air relative humidity, dew point temperature and barometric pressure) and soil variables (pH, total dissolved solids and electrical conductivity) were measured with a Kestrel 5500 Weather Meter and a Lamotte TRACER 1766, respectively. Organic carbon was quantified after by wet digestion method [43], and soil moisture following thermo-gravimetric technique [44].

### 4.2. DNA Extraction and PCR Amplification

DNA was extracted in triplicate, from 250 mg of soil, previously sieved through a 2 mm pore-sized sieve, with DNeasy PowerSoil kit (Qiagen, Hilden, Germany), following the manufacturer’s protocol. The ITS2 nuclear ribosomal region was amplified using Invitrogen Platinum HotStart PCR Master Mix (Thermo Fisher Scientific) with primer fITS7 (5′–GTGARTCATCGAATCTTTG–3′) [46] and ITS4 (5′–TCCTCCGCTTATTGATATGC–3′) [47]. These two primers were added to Illumina overhang adapter sequences: forward overhang: 5′-TCGTCGGCAGCGTCAGATGTGTATAAGAGACAG-[locus specific target primer] and reverse overhang: 5′ GTCTCGTGGGCTCGGAGATGTGTATAAGAGACAG- [locus specific target primer]. The cycling conditions were an initial step at 95 °C for 5 min, 35 cycles at 95 °C for 45 s, 56 °C for 45 s, 72 °C for 45 s and a final extension step of 72 °C for 7 min. The obtained PCR products (c.ca 350 bp) were checked on 1% agarose gel, purified by means of Wizard SV Gel and PCR CleanUp System (Promega), quantified using Qubit 2.0 (Thermo Fisher Scientific, Waltham, MA, USA) and sent to IGA Technology Services S.R.L (Udine, Italy) for Illumina MiSeq platform sequencing (2 × 300 bp).

### 4.3. Bioinformatics and Statistical Analysis

Raw sequence data were processed using the Quantitative Insights Into Microbial Ecology 2 software package (QIIME2 version 2020.6.0) [48]. To avoid poor quality sequences, reads were truncated (>280 bp for forward, >265 bp for reverse reads). The classifier adopted to generate the taxonomic assignment was UNITE community 2020: UNITE QIIME release for Fungi version 20.02.2020. Sequences with ≥97 similarity were assigned to the same operational taxonomic unit (OTU) by means of the QIIME VSEARCH cluster-features-de-novo plugin. The fungal sequencing data were uploaded to the NCBI SRA database under accession number PRJNA779046. The generated dataset and metadata were used to create a *phyloseq* object with the R package “phyloseq” version 1.38 [49]. The total number of reads per sample was rarefied at the lowest sequencing depth to allow statistical comparisons [50]. The OTU table was then rarefied at 1417 sequences per sample by means of the ‘rarefy_even_depth’ function of the R package “phyloseq” [51]. To identify taxa with a relevant role in the fungal communities, the number of samples in which each species occurs was expressed as average prevalence (aP). The occurrence in more than 30% of total samples was the prevalence thresholds defined to identify prevalent species committed to every land use.

With PERMUTEST, the assumption of equal data dispersion among samples was assessed using 999 permutations. A permutational multivariate analysis of variance (PERMANOVA) was performed using 999 permutations to assess the presence of significant differences in the composition of fungal communities (at different taxonomic levels), using abundances of fungal relevant taxa community and alpha diversity indexes (ACE, Shannon (H’), Simpson (D’), Inverse Simpson (1/D’) and Fisher (S’)). To find the differences in the abundance of taxa between contrasting land uses, an exact chi-square test was calculated [52]. Then, non-metric multidimensional scaling plots (NMDS) were carried out to find sample ordination gradients. The NMDS, PERMUTEST and PERMANOVA were performed with R package “vegan” version 2.5 [53], adopting Jaccard’s dissimilarity index as partitioning of variation to assess fungal community composition (presence/absence of OTU) [54]. Gower’s coefficient was adopted to make comparisons based on alpha diversity index as a measure of proximity for mixed data types able to homogenize and scale a set of variables [55,56]. Afterwards, through the function ‘envfit’, the predictor factors were fitted onto the NMDS ordination plot to identify drive factors of changes in the fungal community [57]. The Kruskal–Wallis test was implemented to verify significant differences in soil properties and environmental variables among the three land uses [58].

Log-response ratios (LogRR) were estimated as a measure of size effect exerted by land uses on fungal community parameters [59]. The mean response ratio (MRR) was calculated as the average of LogRR to abundances of individual taxa and alpha diversity index [60]. As sampling variance and standard error of the effect size are not affected by sign changes [61], MRR was expressed as an average to positive LogRR. The assessed features of the fungal community were: (i) abundances of dominant taxa belonging to the taxonomic level, which better explains the differences between land uses, and (ii) values of alpha diversity indexes. The taxonomic level was selected according to higher R^2^ values of PERMANOVA as low R^2^ values of a factor are less effective to explain the differences in community composition than a factor with higher R^2^ [62]. The results observed in samples retrieved in NFA were considered as control groups. LogRR values were estimated with the R package SingleCaseES version 0.4.4 [63]. For each LogRR, confidence intervals (CI) of 95% were computed. If the 95% CI of LogRR did not overlap with 0, then responses were significant at *p* < 0.05 [60]. Finally, unpaired two-sample Wilcoxon test was used to evaluate differences between MMR comparing NFA vs. AGA and NFA vs. MEA.

## 5. Conclusions

The comparison of fungal communities features in this study allows establishing the effect size of land use on soil biodiversity, based on the hypothesis that changes in aspects of fungal communities reflect the severity of land degradation associated with different f-land uses. In the framework of an increasing need to understand the factors driving fertility and microbial diversity of soils, the results revealed how environmental factors and soil properties change under different land uses and alter key fungal components.

The species composition and structure of fungal communities in the assessed Colombian Andosols reveal the richness of soil fungal species compared to similar soil types. The relevant taxa, such as prevalent species and dominant order Hypocreales, Agaricales, and Trichosporonales, were good indicators of land use effects over soil fungal community. Factors such as soil temperature and soil organic matter content were more important drivers than other edaphic factors in determining dissimilarities in order composition among land uses or levels of degradation. Although the response ratio in abundances of prevalent species belonging to dominant orders allowed the identification of deeper changes on soil fungal communities led by driving factors, the stronger response shown by alpha diversity indexes to the level of soil degradation was better at discriminating changes in soil fungal communities based on the type of land use.

## Figures and Tables

**Table 1:** Comparisons of OTU relative abundances at phylum level among land uses.

	NFA ^1^ vs. AGA ^2^		NFA vs. MEA ^3^		AGA vs. MEA	
PHYLUM	*X* ^2*a*^	*p ^b^*	*X* ^2^	*p*	*X* ^2^	*p*
Aphelidiomycota	4.79	0.03	3.91	0.05	0.01	0.92
Ascomycota	150.36	1.0 × (10^−3^)	632.69	1.0 × (10^−3^)	186.44	1.0 × (10^−3^)
Basidiomycota	507.88	1.0 × (10^−3^)	1036.27	1.0 × (10^−3^)	2874.58	1.0 × (10^−3^)
Calcarisporiellomycota	0.01	0.97	0.01	0.94	0.01	0.92
Chytridiomycota	1.66	0.19	0.01	0.94	1.39	0.24
Glomeromycota	41.45	1.0 × (10^−3^)	49.16	1.0 × (10^−3^)	142.65	1.0 × (10^−3^)
Mortierellomycota	419.88	1.0 × (10^−3^)	80.19	1.0 × (10^−3^)	724.42	1.0 × (10^−3^)
Mucoromycota	1212.54	1.0 × (10^−3^)	121.40	1.0 × (10^−3^)	1653.33	1.0 × (10^−3^)
Olpidiomycota	0.01	0.97	0.01	0.94	0.01	0.92
Rozellomycota	6.79	0.01	5.62	0.02	0.01	0.92

^1^ Natural forest areas; ^2^ agricultural activities areas; ^3^ mining extraction activities; *^a^* chi-square statistic; *^b^* *p*-value (significance level ≤ 0.01).

**Table 2:** Prevalent fungal species identified across different land uses in Colombian Andosols.

Land Use	Specie	Average Prevalence (%)	Total Reads
Natural forest areas	*Metarhizium anisopliae*	30.76	1167
	*Saitozyma* sp.	19.23	250
	*Fusarium solani*	15.38	64
	*Trichoderma asperellum*	15.38	549
	*Apiotrichum scarabaeorum*	11.53	603
	*Candida* sp.	11.53	16
	*Clonostachys* sp.	11.53	132
	*Cryptococcus laurentii*	11.53	13
	*Cylindrocarpon* sp.	11.53	201
	*Ganoderma australe*	11.53	75
	*Ganoderma meredithiae*	11.53	18
	*Solicoccozyma terricola*	11.53	55
Agricultural activities areas	*Trichoderma asperellum*	19.23	441
	*Metarhizium anisopliae*	15.38	335
	*Agrocybe pediades*	11.53	13
	*Pholiota abieticola*	11.53	35
	*Suillus cothurnatus*	11.53	28
Mining extraction areas	*Agrocybe pediades*	11.53	11.53

## Data Availability

The datasets generated and analysed during the current study are available from the corresponding author upon publication.

## Supplementary Materials

The following supporting information can be downloaded at: https://www.mdpi.com/article/10.3390/plants12051126/s1, Figure S1. Rarefaction curves showing observed OTU richness in samples of Colombian Andosols; Table S1. ITS2 Sequencing data obtained from Colombian Andosols; Table S1. Rarefaction curves showing observed OTU richness in samples of Colombian Andosols; Table S2: PERMANOVA and PERMUTEST analysis to compare abundances at different taxonomical level and alpha diversity index values in soil fungal communities characterised in Colombian Andosols; Table S3: Average values of environmental and physico-chemical parameters in Colombian Andosols retrieved in the south-eastern region of Antioquia.
