# Supplementary material for: Metabarcoding Reveals Impact of Different Land Uses on Fungal Diversity in the South-Eastern Region of Antioquia, Colombia
Source: Plants (Basel). 2023 Mar 2;12(5):1126. doi: 10.3390/plants12051126 (PMC10005449; doi:10.3390/plants12051126)
Supplement: Supplementary file 1 [file plants-12-01126-s001.zip › plants-2191843-supplementary.pdf]

Supplementary Materials

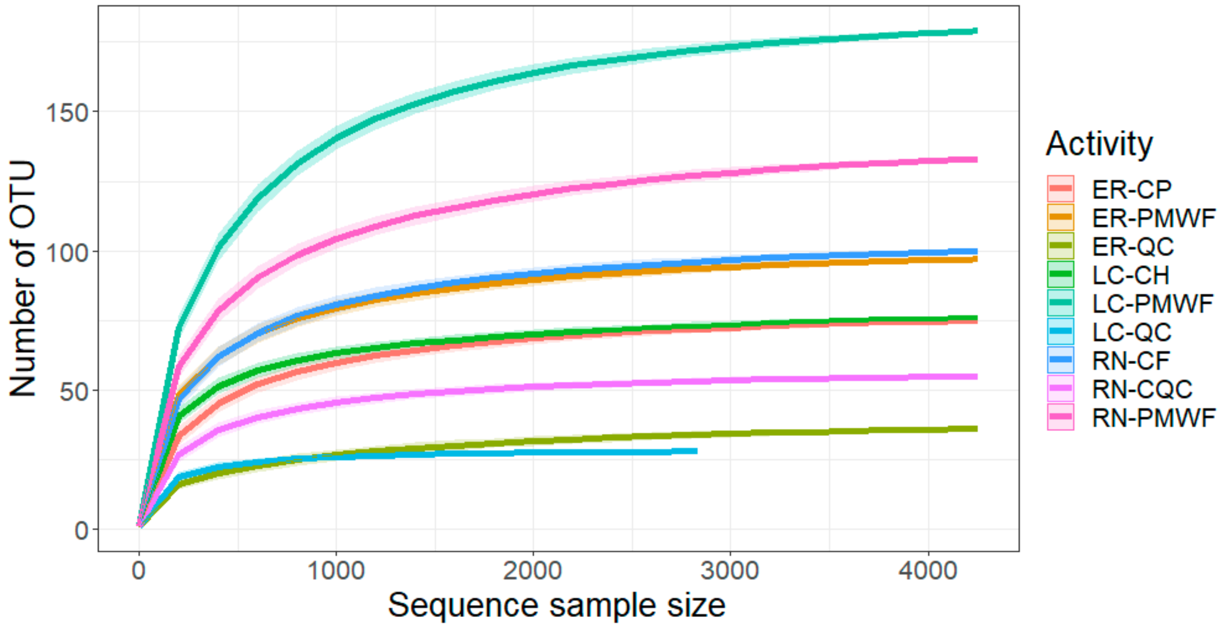

Figure S1. Rarefaction curves showing observed OTU richness in samples taken from El Retiro- Pre-montane wet forest (ER-PMWF); La Ceja- Pre-montane wet forest (LC-PMWF); Rionegro-Pre-montane wet forest (RN-PMWF); El Retiro-Forestry crops of Pinus sp. (ER-CP); La Ceja-Crops of Hydrangea sp.(LC-CH); Rionegro-Crops of Fragaria ananassa (RN-CF); El Retiro-Quarry clays (ER-QC); La Ceja-Quarry clays (LC-QC); Rionegro-Closed quarry clays (RN-CQC).

Table S1. ITS2 Sequencing data obtained from Colombian Andosols.

**Land use / Specific Activity**	**Raw sequences**	**Total valid sequences**	**Average sequences**	**Total OTU observed**
Natural forest areas	**1,414,082**	**105,682**	**11,743**	**489**
**RN- PMWF**[1]	497,376	30,478	10,160	167
**ER- PMWF**[2]	404,467	38,194	12,731	116
**LC- PMWF**[3]	512,239	37,010	12,337	206
Agricultural activities areas	**1,196,336**	**112,374**	**12,486**	**297**
**RN- CF**[4]	334,723	25,434	8,478	120
**ER- CP**[5]	413,927	48,556	16,185	85
**LC- CH**[6]	447,686	38,384	12,795	92
Mining extraction activities	**914,343**	**135,256**	**15,029**	**159**
**RN- CQC**[7]	393,152	45,908	15,303	83
**ER- QC**[8]	262,317	76,191	25,397	45
**LC- QC**[9]	258,874	13,157	4,386	31

[1] Rionegro-Pre-montane wet forest; [2] El Retiro- Pre-montane wet forest; [3] La Ceja- Pre-montane wet forest; [4] Rionegro-Crops of *Fragaria ananassa*; [5] El Retiro-Forestry crops of *Pinus* sp.; [6] La Ceja-Crops of *Hydrangea* sp.; [7] Rionegro-Closed quarry clays; [8] El Retiro-Quarry clays; [9] La Ceja-Quarry clays.

Table S2. PERMANOVA and PERMUTEST analysis to compare abundances at different taxonomical level and alpha diversity index values in soil fungal communities characterized in Colombian Andosols.

Variable	CONTRAST	PERMANOVA		PERMUTEST	
		R^2	P-VALUE	Observed	P-VALUE
**Phylum abundances**	NFA vs. AGA	0.074	0.323	0.523	0.530
	NFA vs. MEA	0.107	0.166	0.780	0.797
	AGA vs. MEA	0.159	0.053	0.672	0.669
**Class abundances**	NFA vs. AGA	0.112	0.049*	0.094	0.099
	NFA vs. MEA	0.158	0.007*	0.068	0.056
	AGA vs. MEA	0.131	0.020*	0.001	0.001*
**Order abundances**	NFA vs. AGA	0.106	0.015*	0.695	0.686
	NFA vs. MEA	0.129	0.010*	0.241	0.232
	AGA vs. MEA	0.094	0.089	0.091	0.078
**Family abundances**	NFA vs. AGA	0.114	0.004*	0.943	0.932
	NFA vs. MEA	0.127	0.004*	0.042	0.035*
	AGA vs. MEA	0.077	0.166	0.002	0.005*
**Genus abundances**	NFA vs. AGA	0.100	0.007*	0.484	0.455
	NFA vs. MEA	0.113	0.006*	0.145	0.119
	AGA vs. MEA	0.076	0.149	0.020	0.024*
**Specie abundances**	NFA vs. AGA	0.097	0.005*	0.306	0.312
	NFA vs. MEA	0.110	0.001*	0.437	0.456
	AGA vs. MEA	0.083	0.075	0.122	0.109
**Alpha diversity index values**	NFA vs. AGA	0.404	0.002*	0.230	0.220
	NFA vs. MEA	0.746	0.001*	0.192	0.186
	AGA vs. MEA	0.580	0.001*	0.883	0.867

* Significance at level ≤0.05

Table S3. Average values of environmental and physico-chemical parameters in Colombian Andosols retrieved in south-eastern region of Antioquia.

Land use/ Specific Activity	Soil temperature (°C)*	Dew point temperature (°C)	Relative air humidity (%)*	Barometric pressure (hPa)	pH	Electrical conductivity (ds/ms)*	Total dissolved solids (ppm)	Moisture (%)*	Organic matter (%)*
**Natural forest areas** (N =9)	**18.28**	**14.87**	**80.71**	**763.80**	**5.43**	**667.78**	**332.15**	**21.28**	**10.56**
**RN- PMWF[1] (N=3)**	18.24	15.20	82.73	743.41	5.37	781.33	407.78	23.20	10.75
**ER- PMWF[2] (N=3)**	18.96	14.96	77.81	769.42	5.73	818.67	405.56	22.48	9.73
**LC- PMWF[3] (N=3)**	17.64	14.45	81.60	778.56	5.18	403.33	183.11	18.16	11.21
**Agricultural Activities Areas** (N =9)	**18.08**	**14.38**	**79.41**	**766.04**	**5.36**	**680.67**	**363.00**	**21.55**	**8.55**
**RN- CF[4] (N=3)**	17.03	13.49	79.99	755.80	5.07	769.00	396.33	23.87	9.11
**ER- CP[5] (N=3)**	17.99	14.23	79.36	774.11	5.73	898.00	453.78	24.28	9.11
**LC- CH[6] (N=3)**	19.21	15.44	78.89	768.22	5.29	375.00	238.89	16.51	7.42
**Mining Extraction Activities** (N =9)	**22.15**	**15.60**	**67.40**	**753.76**	**5.53**	**853.75**	**420.17**	**12.54**	**4.10**
**RN- CQC[7] (N=3)**	20.92	13.94	64.48	736.09	5.54	845.00	404.45	7.68	2.92
**ER- QC[8] (N=3)**	24.48	16.37	61.46	765.91	5.47	876.33	448.89	15.61	5.80
**LC- QC[9] (N=3)**	20.51	16.92	80.70	762.06	5.61	833.00	400.67	15.21	3.33

[1] Rionegro-Pre-montane wet forest; [2] El Retiro- Pre-montane wet forest; [3] La Ceja- Pre-montane wet forest; [4] Rionegro-Crops of *Fragaria ananassa*; [5] El Retiro-Forestry crops of *Pinus* sp.; [6] La Ceja-Crops of *Hydrangea* sp.; [7] Rionegro-Closed quarry clays; [8] El Retiro-Quarry clays; [9] La Ceja-Quarry clays. * Significant differences observed Kruskal-Wallis test ($p<0.05$) among land uses. (RN=Rionegro; ER=El Retiro; LC=La Ceja). (N=Number of samples analized).
